# Coenzyme Q_10_ as an Inhibitor of Effector Release from One-Electron-Reduced Bioreductive Anticancer Prodrugs

**DOI:** 10.3390/molecules30040760

**Published:** 2025-02-06

**Authors:** Robert F. Anderson, Wen Qi

**Affiliations:** 1 Auckland Cancer Society Research Centre, School of Medical Sciences, The University of Auckland, Private Bag 92019, Auckland 1142, New Zealand; 2 School of Chemical Sciences, The University of Auckland, Private Bag 92019, Auckland 1142, New Zealand; 3 Maurice Wilkins Centre for Molecular Biodiscovery, The University of Auckland, Private Bag 92019, Auckland 1142, New Zealand

**Keywords:** tirapazamine, evofosfamide, tarloxotinib, bioreductive prodrug, anticancer, coenzyme Q_10_: electron transfer, pulse radiolysis, kinetics

## Abstract

The kinetic parameters for the release of anticancer effectors from the radical anions of prodrugs through fragmentation have been measured under conditions that model the interfacial region where the enzymatic reduction in the prodrugs takes place. While the back-oxidation of the radical anions via O_2_ mainly occurs under normoxia, preventing radical anion fragmentation, this is not the case for the lower concentrations of O_2_ found in hypoxic regions of tumors. Rate-constant data show that O_2_ concentrations known to bring about a 50% decrease in the level of cell kill arising from the prodrugs in anoxia (the K-value) do not significantly inhibit the fragmentation of radical anions. Evidence is put forward suggesting that radical anions can undergo an electron transfer to ubiquinone (CoQ_10_, UQ) in competition with the fragmentation of the radical anions releasing effectors. The prior inhibition of the synthesis of UQ in cells is put forward as a possible approach to increase the effectiveness of such prodrugs in killing hypoxic tumor cells.

## 1. Introduction

**Figure 1:**
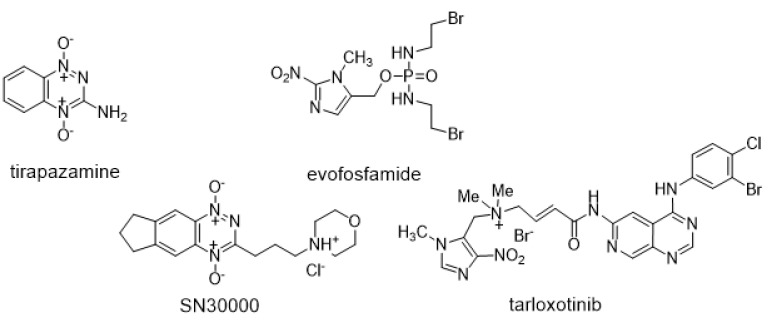
Structures of the prodrugs used in this study.

Dysfunctional vasculature, deregulated cellular metabolism, and the tolerance of low oxygen conditions generate hypoxia in neoplastic tumors [1]. Hypoxia contributes to a poor prognosis across multiple primary tumor sites [2] and also increases resistance to radiotherapy, chemotherapy, and immune-suppressing therapy treatment regimens [3,4,5]. Hypoxia assists disease progression, as outgrowing clones are resistant to apoptosis [6]. This enhances tumor angiogenesis [7], drives the epithelial-to-mesenchymal transition [8], and thus enhances invasion and metastasis [9]. While multiple clinical radiotherapy trials have attempted to harness hypoxic cell radiosensitizers to increase the killing of hypoxic tumor cells, these have shown only a modest benefit [10] and consequently have driven the search for alternative drug-based strategies. Hypoxia-selective bioreductive prodrugs that release potent cytotoxins have been shown in a theoretical study to improve the killing of hypoxic cells over that of radiosensitizers [11]. One such group of prodrugs, shown in Figure 1, utilize one-electron reduction via cellular reductases to release anticancer effectors: evofosfamide, giving rise to DNA cross-links, and the benzotrazine compounds tirapazamine and SN30000, forming cytotoxic radicals or tarloxotinib releasing a kinase inhibitor upon fragmentation of their radical anions [12,13]. Yet, clinical trials either combining the prodrugs with standard anticancer therapies (e.g., tirapazamine with radiotherapy [14] and evofosfamide with doxorubicin [15,16]), and trials with tarloxotinib alone [17,18]), still failed to improve outcomes for patients. However, some single-agent activity for tarloxotinib against the HER2 cohort in a subsequent trial has been seen [19], and evofosfamide combined with gemcitabine in a Phase III pancreatic cancer trial narrowly failed to reach significance [20].

**Figure 2:**
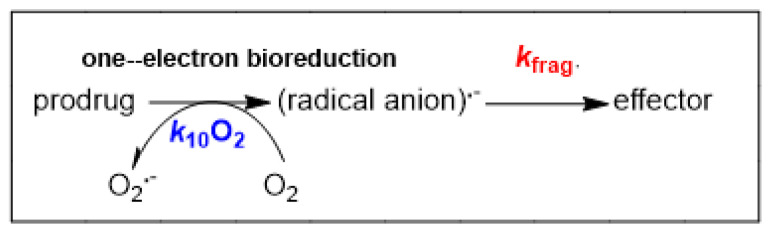
Competitive reactions of the prodrug radical anion. The parameter *k*_10_O_2_ is the rate constant for O_2_ reacting with the prodrug radical anion; *k*_frag._ is the rate constant for the release of the effector from the prodrug radical anion.

The cytotoxicities of the above bioreductive prodrugs are inhibited under aerobic conditions (21% O_2_) and assumed to be mainly so under normoxia [13] (ca. average of 6.1% O_2_ in peripheral tissues) [21], with the drugs becoming highly cytotoxic in low levels of oxygen (ca. 0.02- 1% O_2_). The concentration of oxygen ([O_2_]) that halves the cytotoxicity level of the prodrugs seen in anoxia, known as the K-value, ([O_2_]^K^), varies widely from tirapazamine and its related analog SN30000 at 1.21 μM and 1.14 μM [O_2_]^K^, respectively [22,23], to evofosfamide at 0.27 μM [O_2_]^K^ [24] and 0.034 μM [O_2_]^K^ for tarloxotinib [25]. It is generally thought that the concentration levels of oxygen found in normal tissues suppress the release of the effectors from the radical anions via back-oxidation through an electron transfer to O_2_ [13]. In kinetic terms, the pseudo-first-order rate-constant derived from ***k*_10_O_2_**/M^−1^ s^−1^ and multiplied by the K-value [O_2_]^K^ /M competes with the first-order release of the effectors via fragmentation, ***k*_frag._**/s^−1^ (Figure 2).

The one-electron bioreduction in electron-affinic prodrugs has been identified to occur through various flavoproteins, such as P450 oxidoreductase (POR) in the endoplasmic reticulum [26,27], at the cell surface through STEAP4 [28], and through the mitochondrial electron transport chain [29] to form the radical anions of the prodrugs. While ***k*_10_O_2_** is governed by the difference in one-electron reduction potentials (*E*^0′^) between the prodrug and O_2_ [30,31], ***k*_frag_**. is dependent on the respective prodrug and leaving effector structures. Kinetic data for the back-oxidation of the prodrug radical anions, obtained by utilizing pulse radiolysis [32], enable a comparison of the pseudo first-order rate constants at O_2_ concentrations predicting a 50% decrease in anoxic cytotoxicity (the K-value; Table 1) with ***k*_frag._**

Theoretically, both first-order rate constants should be similar at an O_2_ concentration equal to the K-value, [O_2_]^K^; however ***k*_frag_**./s^−1^ substantially out-competes ***k*_10_O_2_** × [O_2_]^K^/s^−1^, shown in Table 1; i.e., the cytotoxicity should be greater than that observed, approaching that found in anoxia. What are the possible reasons for such a mismatch between the above kinetic analysis and the predicted cytotoxicity of the bioreductive prodrugs in hypoxic cells?

Does hypoxia selectivity arise from O_2_ modifying the released effectors, rather than the back-oxidation of radical anion? This might play a minor role for tirapazamine and SN30000, which release cytotoxic radicals, some of which may react with O_2_. However, evofosfamide and tarloxotinib release a cytotoxic mustard and a kinase inhibitor, respectively; the reactivity of both of these effectors is unaffected by O_2_.

Does hypoxia selectivity arise from O_2_ intercepting the reducing equivalents, e.g., on the flavin reductase proteins and FMN in Complex I of the mitochondrial electron transport chain? This, too, seems unlikely, as the oxidation of the reduced flavin (the active reductant moiety, also in POR, which, when fully reduced, is a singlet) and its semiquinone via O_2_ (a triplet) proceed very slowly with rate constants of ca. 250 M^−1^ s^−1^ and ~10^4^ M^−1^ s^−1^ [37].

**Figure 3:**
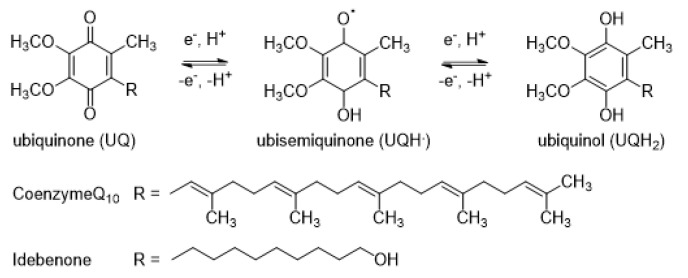
Structures of ubiquinone/ubisemiquinone/ubiquinol.

We are left with the possibility of an electron acceptor other than O_2_ that oxidizes the radical anions of the prodrugs, inhibiting the full release of the effectors. It is likely that such an oxidant is in the vicinity of where the reduction in the prodrugs takes place and that it possesses an *E*^0′^ similar to or higher than that of O_2_. The POR protein is embedded in the membrane of the endoplasmic reticulum and the inner membrane of mitochondria [38], with a reduction in water-soluble drugs taking place at the interface between the membrane and cytosol. The *E*^0′^ of O_2_ in such an interfacial region will be between that of −155 mV in aqueous solution [39] and ca. −630 mV in aprotic membranes [40]. A likely candidate is lipophilic ubiquinone (CoQ_10_, UQ), shown in Figure 3, which is present in all membranes of mammalian cells in equilibrium with its fully reduced form, ubiquinol (UQH_2_). UQ has an *E*^0′^ of ca. −420 mV in aprotic solvents (for the ubiquinone analogs idebenone and menaquinone) [41,42], while a water-soluble analog, mitoQ, has an *E*^0′^ of −105 mV [41]. These *E*^0′^ values are higher than those for O_2_ in both mediums. The polyisoprenoid chain of CoQ_10_ is present in the central hydrophobic region between the phospholipid double layer, while the redox active benzoquinone group is located on the outer or inner surface of the membrane [43,44]. The combined concentration of UQ + UQH_2_ in human plasma is ca. 1 μM [45,46], with the concentration of UQ in normoxia being 50–100 nM [47]. The UQ/(UQ + UQH_2_) ratio decreases in hypoxia, reaching a high conversion to UQH_2_ in acute hypoxia [48]. The UQ + UQH_2_ concentration in human tissues varies between 8 mg/g in the lung and 114 mg/g in the heart [49], with their subcellular location being mainly in the mitochondria [44]. The concentration in plasma membranes of 0.7 mg/mg protein [44] is ca. 1 μM, of which only ca. 10% (100 nM) is the oxidized form, UQ [50]. In this study, we investigate a possible role for the one-electron reduced forms of the prodrugs undergoing an electron transfer to UQ in competition to their release of effectors.

An important consideration is factors controlling the one-electron transfer from prodrug radical anions to oxidants. A reduction in the prodrugs occurs via membrane-embedded reductases in the interfacial space between aqueous and lipid forms, where the formed radical anions of the prodrugs undergo fragmentation to release effectors in competition to electron-transfer to O_2_. The prodrugs contain lipophilic aromatic moieties that may aid their partial solubility in membranes and effectively enable two-dimensional diffusion to the reduction sites on the proteins. Alternatively, an outer-sphere electron transfer over a short distance takes place in the interfacial region. This region is characterized by the rise in the dielectric constant from a lipid-type environment (ε_r_ = 2.02; for example, *n*-hexane) to water (ε_r_ = 78.4) over ca. 10 nm [51]. Hence, it can be argued that relevant electron-transfer kinetic studies should be undertaken in systems that model the interfacial environment. We chose methanol as the solvent in which to investigate electron-transfer reactions between the prodrugs and UQ, as (i) methanol has an ε_r_ = 33, which is intermediate between lipid and water, and (ii) radiation chemistry studies have shown that the ionization of methanol exclusively produces reducing species (solvated electron, e-, and hydroxymethyl radical, •CH_2_OH) [52].

## 2. Results

### 2.1. Radiolysis Studies in Methanol

The radiolytically produced solvated electron in methanol, e^−^, reacts with electron-affinic prodrugs (D) and UQ at near diffusion-controlled rates (*k*_1_, *k*_2_ ~10^10^ M^−1^ s^−1^) to form radical anions of the prodrugs, (D•^−^) and ubisemiquinone (UQ•^−^), which can undergo spontaneous protonation via the protic solvent [53]. The •CH_2_OH radical reacts with UQ via an electron transfer with a rate constant, *k*_3_, of 1.1–1.4 × 10^9^ M^−1^ s^−1^ [54], and with nitroaromatic compounds, *k*_4_, some one to two orders of magnitude slower, forming weakly absorbing adducts on the nitro group that are stable in methanol for several milliseconds [55]. Hence, to study possible electron-transfer reactions from D•^−^ to UQ, *k*_6_, in competition with the fragmentation of the prodrug radical anions to release effectors, ***k*_frag_**., it is necessary to form D•^−^ through scavenging the e- by employing [prodrug] >> [UQ]. The UQ compounds CoQ_10_ and the more methanol-soluble analog idebenone were found to be reduced via the prodrug radical anions, (D•^−^), to form ubisemiquinone (UQH•), followed by its known deprotonation in methanol, reaction 7 [54], during the electron-transfer reaction. The •CH_2_OH radical reacts with both UQ and the prodrugs as side reactions, producing an initial amount of UQH• and adducts that do not interfere with the analysis of a subsequent electron transfer from D•^−^ to UQ.

CH_3_OH ^^^^^ → e^−^ + (CH_3_OH^+^) → •CH_2_OH + H^+^

e^−^ + D → D•^−^/DH• (1)

e^−^ + UQ → UQ•-/UQH• (2)

•CH_2_OH + UQ → UQ•^−^/UQH• + CH_2_O (3)

•CH_2_OH + D → (HOCH_2_D•) → D•^−^/DH• + CH_2_O_._ (4)

DH• → effector + (D)′• ***k*_frag_** (5)

D•^−^/DH• + UQ → D + UQ•^−^/UQH• ***k_6_*** (6)

UQH• → UQ•^−^ + H^+^ (7)

### 2.2. One-Electron Reduction in UQ via Prodrug Radical Anions

**Figure 4:**
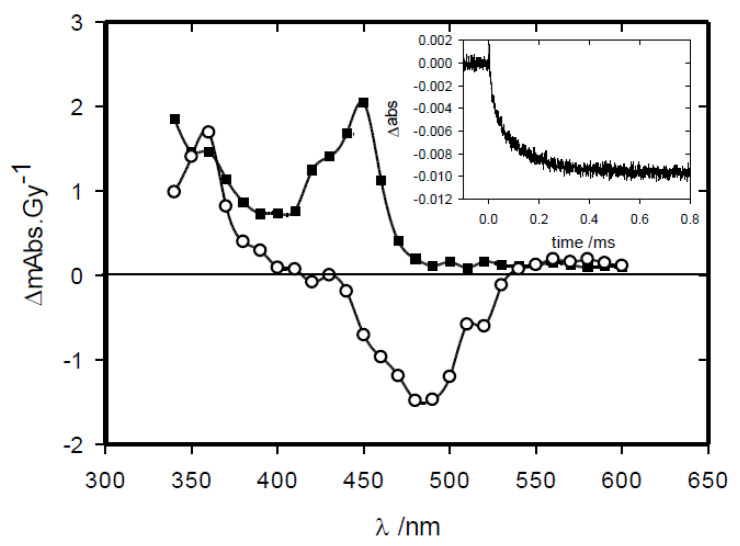
Difference spectra of one-electron reduced forms of tirapazamine (300 μM) and idebenone (40 μM) in N_2_-saturated methanol following pulse radiolysis (5.2 Gy in 200 ns), ○ 2 μs, ■ 400 μs after the pulse. Insert: transient of transmittance against time at 430 nm.

**Figure 5:**
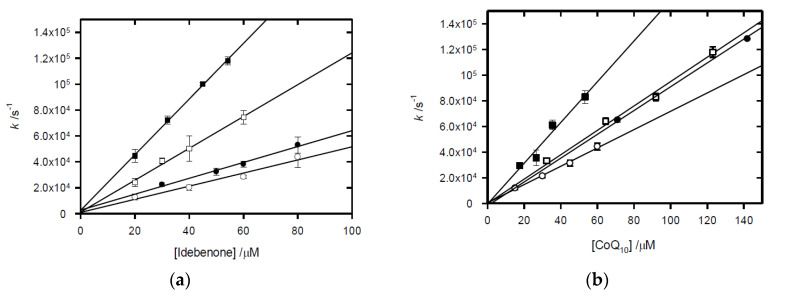
(**a**) Dependence of the observed rate constant at 430 nm for the formation of the ubisemiquinone of idebenone on the concentration of idebenone following the pulse radiolysis of N_2_-saturated methanol solutions containing ○ tirapazamine (300 μM), ● tarloxotinib (300 μM), □ SN30000 (275 μM) and ■ evofosfamide (1 mM). (**b**) Dependence of the observed rate constant at 430 nm for the formation of the ubisemiquinone of CoQ_10_ on the concentration of CoQ_10_ following the pulse radiolysis of N_2_-saturated methanol solutions containing ○ tirapazamine (300 μM), ● tarloxotinib (300 μM), □ SN30000 (275 μM), and ■ evofosfamide (1 mM).

Example spectra and a time-resolved transient observed for the reduction in idebenone via one-electron reduced tirapazamine are presented in Figure 4. The initial difference spectrum displays characteristics one-electron reduced tirapazamine [33,56], and the second spectrum features match those of ubisemiquinone (UBH•) [54]. The observed absorption of 2.05 × 10^−3^ Gy^−1^ corresponds to a radical yield of 0.315 μM. Gy^−1^ (μmol J^−1^) when an extinction coefficient of ca. 6500 M^−1^ cm^−1^ at 450 nm is used [54]. This observation is consistent with a minor amount of the •CH_2_OH radical species being scavenged via idebenone (≥0.035 μM.Gy^−1^) and the e- (≤0.28 μM Gy^−1^) being scavenged via tirapazamine, followed by an electron transfer to idebenone. The second-order rate constants of the electron transfer for all four compounds were determined from the plot of observed first-order rate constants for increasing concentrations of idebenone, shown in Figure 5a, with data presented in Table 2. Similar studies were carried out with CoQ_10_ as the UQ oxidant of one-electron reduced forms of the prodrugs, yielding plots of the first-order rate constants against [CoQ_10_], shown in Figure 5b, from which the second-order rate constants are presented in Table 2.

### 2.3. Rate Constants of Fragmentation of Prodrug Radical Anions

**Figure 6:**
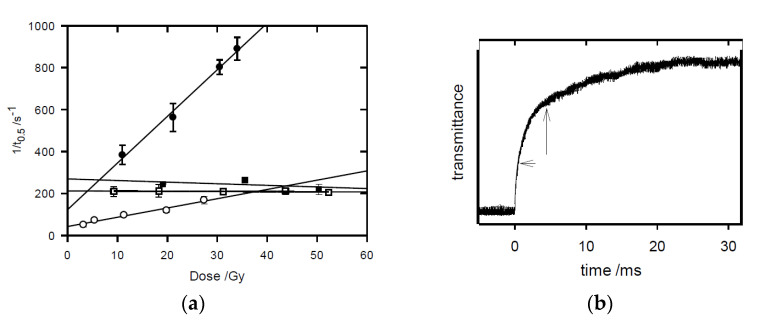
(**a**) Dependence of the reciprocal of the first half-life of radicals on the initial radical concentration formed with an increasing radiation dose. Radical anions of ○ tirapazamine, ● tarloxotinib, □ SN30000, and ■ evofosfamide were produced in N_2_-saturated methanol. (**b**) Example transient for SN30000 showing initial transmittance, followed by a t_0.5_ measurement of the mixed-order transient.

Values for the rate constant of fragmentation of the prodrugs following their one-electron reduction in irradiated methanol, *k*_frag.,_ were obtained by measuring the *t*_0.5_ values of transients over a range of pulsed radiation doses for each of the prodrugs, shown in Figure 6, from which a linear regression of the data yielded the *k*_frag._ values calculated from the intercept of the plots. The obtained values are presented in Table 2.

### 2.4. Rate Constants for the Oxidation of Prodrug Radical Anions via O_2_

The second-order rate constants for the oxidation of the radical anions of the prodrugs via O_2_ in methanol, ***k*_20_O_2_,** determined from plots of the first-order decay of their transients against increasing [O_2_] (see Supplementary Materials), together with electron-transfer rate constants of *k_6_* × [UQ] (for [UQ] = 100 nM) and ***k*_20_O_2_**× K-value (Table 1), are presented in Table 2 for a comparison with *k*_frag._ in methanol.

## 3. Discussion

Although biological electron transfers mainly occur in or close to membranes, there are few experimental systems that adequately model the interfacial region between a membrane and the bulk solution. Reverse micelles in aqueous solution have been used to study the electron transfer between viologen radicals and quinones [57]; however, such a system could not be used here, as the UQs would be severely diluted in the organic solvent. This study modeled the effect of a change in the dielectric constant on electron-transfer reactions, a feature of the interfacial region. Electron-transfer reactions of the prodrug radical anions compete with the breakdown of the radical anions to release their masked effectors. It is seen that ***k*_frag_**./s^−1^ out-competes the back-oxidation of the radical anions of the prodrugs via O_2_, in both aqueous and methanol solutions, at [O_2_], where 50% of the toxicity in anoxia is expected (the K-values/M). Such a model requires an additional mechanism of electron transfer in competition with ***k*_frag_**. The evidence put forward in this study suggests an electron transfer from the prodrug radical to UQ via an outer-sphere electron transfer, rather than through the prior sequestration of the prodrugs into the membrane of cells. This analysis must be considered qualitative, as UQ + UQH_2_ levels in plasma membranes, for example (ca. 6% of cell content) [49], will vary with the cell type and in cancer tissues, which have a ca. 50% lower concentration than non-cancerous tissues [58,59]. It could well be that the UQ concentrations in HCT116 and FaDu cells are less than 100 nM used in the kinetic comparisons in Table 2, while the UQ concentration in HT29 cells is higher. Nevertheless, this study supports a possible role for UQ as an antagonist in the effectiveness of bioreductive prodrugs that are capable of forming hypoxia-targeted effectors following their one-electron bioreduction. Notably, work with isogenic ρ^0^ cells, which lack respiring mitochondria and consequently UQ, exhibit much greater sensitivity to prodrugs than normal (mitochondria-containing) cells [29]. Another outcome from this study is to question the long-held exclusive role that O_2_ plays in fostering the selectivity of these prodrugs to act in hypoxia.

At normoxic levels of O_2_ in tissues (≥6% O_2_, [O_2_] ca. 78 μM), the electron transfer from the prodrug radical anions to both O_2_ and UQ needs to be considered in relation to *k*_frag_. This can be expressed as a ‘kinetic ratio’ for protection from the release of cytotoxic effectors under normoxic conditions, which is shown in Table 3.

**Figure 7:**
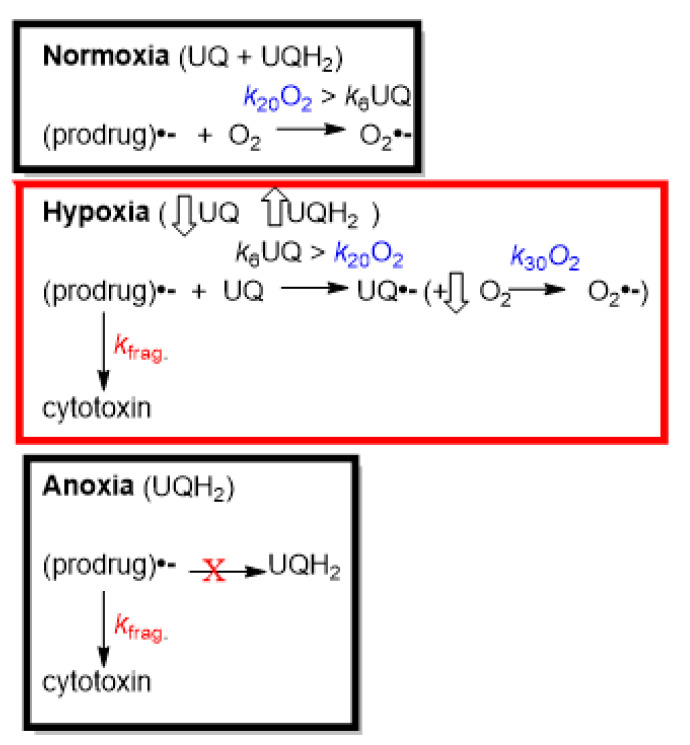
Interaction of UQ and O_2_ with the radical anions of prodrugs. Suboptimal release of cytotoxin from prodrug radical anions in hypoxia due to competing electron transfer to UQ.

The combined back-oxidation of the radical anions via O_2_ in normoxia and the electron transfer to CoQ_10_ from the radical anions of evofosfamide and tarloxotinib out-compete the release of their cytotoxins to a large extent. However, the single-agent activity seen for evofosfamide in tumors has been accounted for via a significant bioreduction in evofosfamide in oxic regions of the tumors [24]. The above analysis for tirapazamine and SN30000 also predicts a greater release of cytotoxins from their radical anions in normoxia, which may be partially responsible for some of the cytotoxicity of normal tissues. This might well be related to the benzotrazine di-*N*-oxide structures of tirapazamine and SN30000, as opposed to the nitroimidazole structures of evofosfamide and tarloxotinib. As the rate constants for the electron transfer from any such formed ubisemiquinone to O_2_ are ca. two orders of magnitude greater than for the radical anions of aromatic nitro and di-*N*-oxide compounds to O_2_ [31] (***k*_30_O_2_** >> ***k*_20_O_2_**, Figure 7), superoxide (O_2_•^−^) still results. (In reality, an equilibrium between UQH•/O_2_ and UQ/O_2_•^−^ occurs but will be unstable as O_2_•^−^ is removed via, e.g., superoxide dismutase.)

Our hypothesis is that it is the combination of UQ and O_2_ that protects normal well-oxygenated tissues from the release of cytotoxins from prodrugs, with O_2_ taking the electron off both the radical anion of the prodrugs and ubisemiquinone (UQH•) There is a marked difference between the average O_2_ concentration in normal tissues (ca. 6%) and tumors (ca. 1.4%), with the hypoxic fraction of cells in tumors likely having O_2_ concentrations lower than this average. Normal (well-oxygenated) tissues are unlikely to become sensitive to the prodrugs following prior treatment with statins to decrease the CoQ_10_ concentration in cells due to the low concentration of statins in plasma (ca. ≤ 15 nM) [60]. It is unknown whether this low concentration of statins in hypoxic cells could effect a decrease in the resistance to toxicity. There are other possible electron acceptors in the cell, such as cytochrome c (cyt c). In previous work, it has been observed that evofosfamide altered cyt c spectra [29]. However, cyt c is downstream of UQ in the electron transport system, so it will be impacted by default.

In conclusion, rather than an exclusive reaction occurring between the radical anions of prodrugs and O_2_ levels found in oxic cells, UQ may play a significant part in suppressing the oxic toxicity of such drugs. O_2_ acts as a surrogate in maintaining the UQ/(UQ + UQH_2_) ratio, and upon a decrease in the concentration of UQ as the concentration of O_2_ falls, the release of cytotoxins is predicted to be increased. In prolonged anoxia, when UQ is majorly converted to UQH_2_, the unimpeded release of cytotoxins from the prodrugs occurs through bioreduction, resulting in maximum cytotoxicity. In normoxia, prodrugs based on the benzotriazine di-*N*-oxide structure may exhibit an undesirable greater release of cytotoxins in normal tissues than prodrugs of nitroimidazole structure.

## 4. Materials and Methods

### 4.1. Compounds

Tirapazamine, SN30000, and tarloxotinib were synthesized at the Auckland Cancer Society Research Centre, as reported previously. Evofosfamide was a gift from Threshold Pharmaceuticals, CA. Coenzyme Q_10_ (Sigma, St. Louis, MO, USA) and idebenone (Combi-Blocks, San Diego, CA, USA) were used as supplied. Reagent-grade methanol (99.9%) was obtained from Merck (Rahway, NJ, USA).

### 4.2. Radiation Chemistry

Time-resolved pulse radiolysis studies with the prodrugs (0.25–1 mM) were carried out using the University of Auckland’s facility. Details of the 4 MeV linear accelerator, the optical radical detection system, and the method of dosimetry used have been published [61]. Methanol solutions were purged with N_2_ for 20 min before pulse radiolysis. Absorption spectra are presented as the difference between the produced radicals and the preirradiated compounds. Kinetic measurements of the reactions between the radical anions of the prodrugs and UQ and O_2_ are the average of at least 3 determinations for increasing concentrations of the prodrugs and saturating gas mixtures of N_2_/O_2_, with the solubility of O_2_ in methanol of 10.3 mM at 25 °C taken into account [62].

The decay of the radical anions in the absence of UQ, with the initial concentration [C]_0_, was by a mixed 1st-order process, *k*_1_ (***k*_frag_**_._), and a second-order process, *k*_2_ (bimolecular, radical–radical), which can be related to the half-life of the radical anion, *t*_0.5_, via exp(*k*_1_.*t*_0.5_) = (2*k*_1_ + *k*_2_[C]_0_)/(*k*_1_ + *k*_2_[C]_0_). When [C] = 0, that is, the intercept of a plot 1/*t*_0.5_ vs. [C], *k*_1_ = ln0.5(1/*t*_0.5_), i.e., giving ***k*_frag_**_._ values_._

## Figures and Tables

**Table 1:** Kinetic comparison of back-oxidation and effector release in aqueous solution.

Prodrug	Cell Line	K-Value 10^6^[O_2_]^K^/M	10^−6^*k*_10_O_2_ /M^−1^ s^−1^	*k*_10_O_2_ ×10^6^[O_2_]^K^/s^−1^	*k*_frag_./s^−1^
tirapazamine	HT29	1.21 ± 0.09 [29]	6.20 ± 0.25 [33]	7.5	112 ± 23 [34]
SN30000	HTs9	1.14 ± 0.24 [35]	3.33 ± 0.03 [35]	3.8	125 ± 15 [35]
evofosfamide	HCT116	~0.27 [24]	3.27 ± 0.18 [36]	~0.88	32 ± 2 [36]
tarloxotinib	FaDu	0.034 ± 0.020 [25]	5.92 ± 0.58 ^2^	0.20	20 ± 5 ^1^

^1^ This study’s Figure S1; ^2.^ Figure S2.

**Table 2:** Kinetic comparison of electron transfer rate constants in methanol and effector release.

Prodrug D	10^−6^*k*_20_O_2_ (DH• + O_2_) ^1.^ /M^−1^ s^−1^	10^−8^*k*_6_ (DH• + Idebenone) /M^−1^ s^−1^	10^−8^*k*_6_ (DH• + CoQ_10_) /M^−1^ s^−1^	*k*_20_O_2_ × 10^6^[O_2_]^K^ /s^−1^	*k*_6_ × [Idebenone] ^2.^ /s^−1^	*k*_6_ × [CoQ_10_] ^2.^ /s^−1^	*k*_frag._ /s^−1^
tirapazamine	1.17 ± 0.07	5.06 ± 0.67	7.15 ± 0.40	1.4 ± 0.1	51 ± 7	72 ± 5	29 ± 5
SN30000	2.46 ± 0.44	12.3 ± 0.7	9.50 ± 0.21	2.8 ± 0.6	123 ± 7	91 ± 6	102 ± 2
evofosfamide	24.1 ± 3.1	21.5 ± 0.5	15.9 ± 2.3	~6.5 ± 0.8	215 ± 13	153 ± 12	186 ± 30
tarloxotinib	24.3 ± 1.6	6.13 ± 0.48	8.35 ± 0.15	0.8 ± 0.5	61 ± 5	84 ± 1	85 ± 25

^1.^ see Figures S2–S5 ^2.^ [ ] = 100 nM.

**Table 3:** Kinetic comparison of combined electron transfer from prodrug radical anions to O_2_ and CoQ_10_ with *k*_frag_.

Prodrug D	10^−6^*k*_20_O_2_ (DH• + O_2_) × 78 μM/s^−1^	10^−8^*k*_6_ (DH• + CoQ_10_) × 100 nM/s^−1^	*k*_frag._ /s^−1^	Kinetic Ratio (*k*_20_O_2_ + *k*_6_^′^)/*k*_frag._
tirapazamine	91	72	29	5.62
SN30000	192	95	102	2.81
evofosfamide	1880	159	186	11.0
tarloxotinib	1895	84	85	23.3

## Data Availability

The original contributions presented in this study are included in the article/Supplementary Material. Further inquiries can be directed to the corresponding author.

## Supplementary Materials

The following supporting information can be downloaded at https://www.mdpi.com/article/10.3390/molecules30040760/s1, Figure S1: Determination of *k*_frag_. for the radical anion of tarloxotinib in water; Figure S2: Rate constant for the reaction of the radical anion of tarloxotinib with O_2_ in water; Figure S3: Rate constant for the reaction of the radical anion of tirapazamine with O_2_ in methanol; Figure S4: Rate constant for the reaction of the radical anion of SN30000 with O_2_ in methanol; Figure S5: Rate constant for the reaction of the radical anion of evofosfamide with O_2_ in methanol; Figure S6: Rate constant for the reaction of the radical anion of tarloxotinib with O_2_ in methanol.
