# Correction: Anderson, R.F.; Qi, W. Coenzyme Q_10_ as an Inhibitor of Effector Release from One-Electron-Reduced Bioreductive Anticancer Prodrugs. *Molecules* 2025, *30*, 760

**DOI:** 10.3390/molecules30173515

**Published:** 2025-08-28

**Authors:** Robert F. Anderson, Wen Qi

**Affiliations:** 1 Auckland Cancer Society Research Centre, School of Medical Sciences, The University of Auckland, Private Bag 92019, Auckland 1142, New Zealand; 2 School of Chemical Sciences, The University of Auckland, Private Bag 92019, Auckland 1142, New Zealand; wqi066@aucklanduni.ac.nz; 3 Maurice Wilkins Centre for Molecular Biodiscovery, The University of Auckland, Private Bag 92019, Auckland 1142, New Zealand

In the original publication [1], there was some mistakes in Tables 2 and 3. Unfortunately, the proof for the *Molecules* paper occurred when one author was away on summer vacation when they did not have office facilities, meaning that checking proved to be inadequate. 

These corrections do not materially change the conclusions derived from the table. This correction was approved by the Academic Editor. The original publication has also been updated.

The corrected Table 2 and Table 3 appear below.

**Table 2:** Kinetic comparison of electron transfer rate constants in methanol and effector release.

Prodrug D	10^−6^*k*_20_O_2_ (DH• + O_2_) ^1.^ /M^−1^ s^−1^	10^−8^*k*_6_ (DH• + Idebenone) /M^−1^ s^−1^	10^−8^*k*_6_ (DH• + CoQ_10_) /M^−1^ s^−1^	*k*_20_O_2_ × 10^6^[O_2_]^K^ /s^−1^	*k*_6_ × [Idebenone] ^2.^ /s^−1^	*k*_6_ × [CoQ_10_] ^2.^ /s^−1^	*k*_frag._ /s^−1^
tirapazamine	1.17 ± 0.07	5.06 ± 0.67	7.15 ± 0.40	1.4 ± 0.1	51 ± 7	72 ± 5	29 ± 5
SN30000	2.46 ± 0.44	12.3 ± 0.7	9.50 ± 0.21	2.8 ± 0.6	123 ± 7	91 ± 6	102 ± 2
evofosfamide	24.1 ± 3.1	21.5 ± 0.5	15.9 ± 2.3	~6.5 ± 0.8	215 ± 13	153 ± 12	186 ± 30
tarloxotinib	24.3 ± 1.6	6.13 ± 0.48	8.35 ± 0.15	0.8 ± 0.5	61 ± 5	84 ± 1	85 ± 25

^1.^ see Figures S2–S5 ^2.^ [ ] = 100 nM.

**Table 3:** Kinetic comparison of combined electron transfer from prodrug radical anions to O_2_ and CoQ_10_ with *k*_frag_.

Prodrug D	10^−6^*k*_20_O_2_ (DH• + O_2_) × 78 μM/s^−1^	10^−8^*k*_6_ (DH• + CoQ_10_) × 100 nM/s^−1^	*k*_frag._ /s^−1^	Kinetic Ratio (*k*_20_O_2_ + *k*_6_^′^)/*k*_frag._
tirapazamine	91	72	29	5.62
SN30000	192	95	102	2.81
evofosfamide	1880	159	186	11.0
tarloxotinib	1895	84	85	23.3
