# Supplementary material for: Coenzyme Q10 as an Inhibitor of Effector Release from One-Electron-Reduced Bioreductive Anticancer Prodrugs
Source: Molecules. 2025 Feb 6;30(4):760. doi: 10.3390/molecules30040760 (PMC11858625; doi:10.3390/molecules30040760)
Supplement: Supplementary file 1 [file molecules-30-00760-s001.zip › molecules-3361860-supplementary.pdf]

Supplementary Materials

Coenzyme Q_{10} as an Inhibitor of Effector Release from one-Electron-Reduced Bioreductive Anticancer Prodrugs

Robert F. Anderson and Wen Qi

Contents Pages

A. Determination of *k*frag. for the radical anion of tarloxotinib.in water 1
B. Rate constant for the reaction of the radical anion of tarloxotinib with O_2 in water 2
C. Rate constant for the reaction of the radical anion of tirapazamine with O_2 in methanol 2
D. Rate constant for the reaction of the radical anion of SN30000 with O_2 in methanol 3
E. Rate constant for the reaction of the radical anion of evofosfamide with O_2 in methanol 3
F. Rate constant for the reaction of the radical anion of tarloxotinib with O_2 in methanol 4

A. Determination of *k*frag. for the radical anion of tarloxotinib.in water

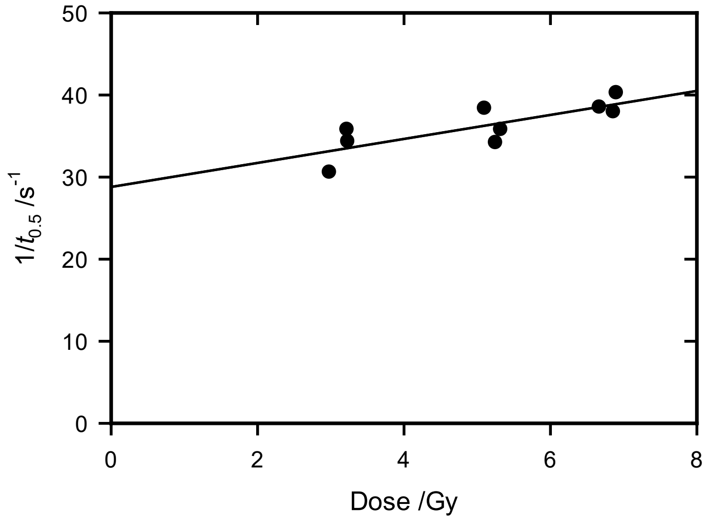

Figure S1. Dependence of the reciprocal of the first half-lifes of transients on radiation dose observed at 420 nm following pulse radiolysis of tarloxotinib (1 mM), sodium formate (0.1 M) and sodium phosphate (2.5 mM) at pH 7 in N_2-saturated water.

B. Rate constant for the reaction of the radical anion of tarloxotinib with O_2 in water

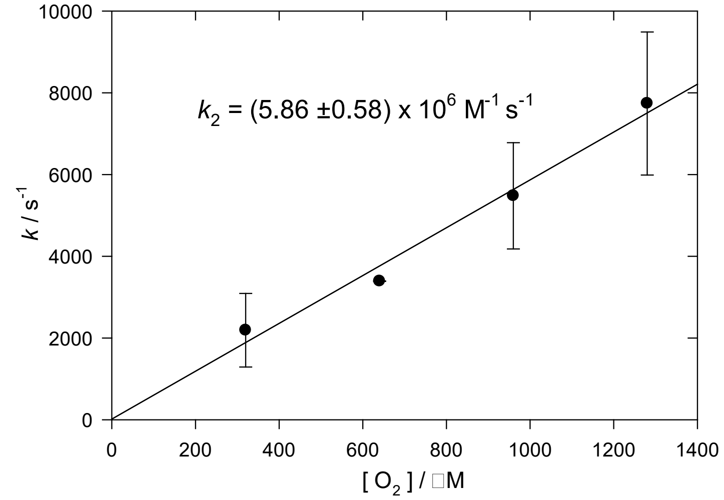

Figure S2. Dependence of the 1st-order rate constants for the decay of the radical anion of tarloxotinib observed at 440 nm on the concentration of O_2 following pulse radiolysis (ca. 10 Gy in 200 ns) of solutions saturated with O_2/N_2O mixtures, containing tarloxotinib (1 mM), sodium formate (0.1 M) and sodium phosphate (2.5 mM) at pH 7 (●) (The 2nd-order rate constant, k_2, is derived from the slope of the graph).

C. Rate constant for the reaction of the radical anion of tirapazamine with O_2 in methanol

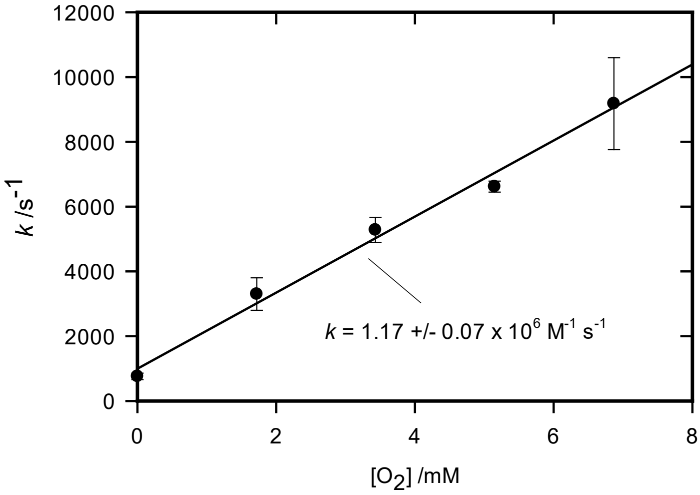

Figure S3. Dependence of the 1st-order rate constants for the decay of the radical anion of tirapazamine observed at 360 nm on the concentration of O_2 following pulse radiolysis (ca. 10 Gy in 200 ns) of solutions saturated with O_2/N_2 mixtures, containing tirapazamine (1 mM) in methanol (●).

D. **Rate constant for the reaction of the radical anion of SN30000 with O_2 in methanol**

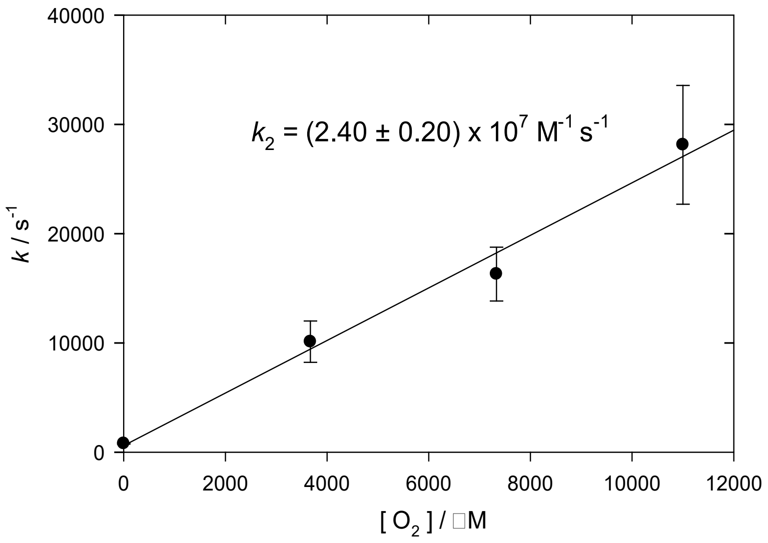

Figure S4. Dependence of the 1st-order rate constants for the decay of the radical anion of SN30000 observed at 360 nm on the concentration of O_2 following pulse radiolysis (ca. 10 Gy in 200 ns) of solutions saturated with O_2/N_2 mixtures, containing SN30000 (1 mM) in methanol (●).

E. **Rate constant for the reaction of the radical anion of evofosfamide with O_2 in methanol**

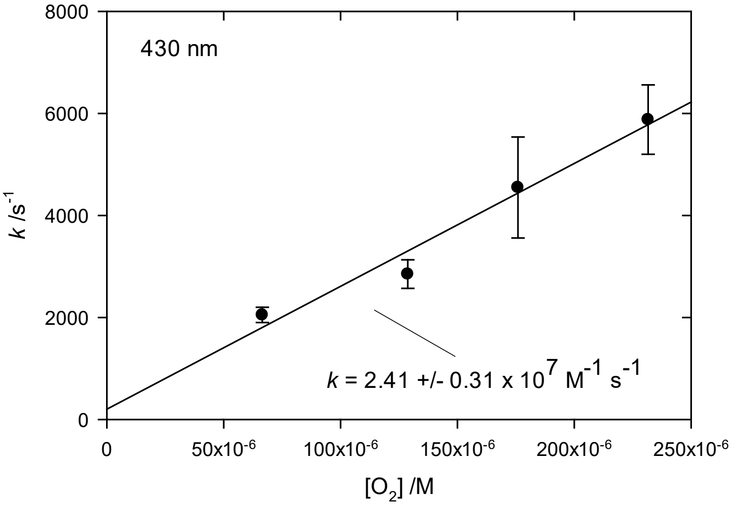

Figure S5. Dependence of the 1st-order rate constants for the decay of the radical anion of evofosfamide observed at 440 nM on the concentration of O_2 following pulse radiolysis (ca. 10 Gy in 200 ns) of solutions saturated with O_2/N_2 mixtures, containing evofosfamide (1 mM) in methanol (●).

F. Rate constant for the reaction of the radical anion of tarloxotinib with O_2 in methanol

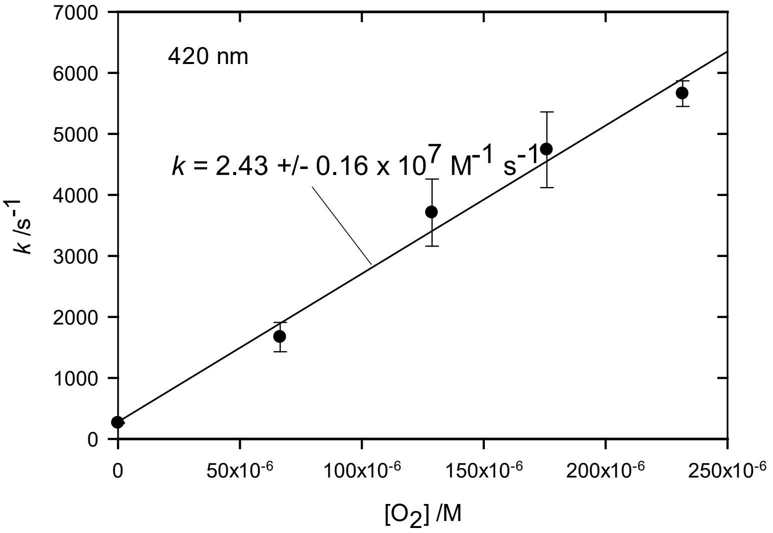

Figure S6. Dependence of the 1st-order rate constants for the decay of the radical anion of tarloxotinib observed at 420 nm on the concentration of O_2 following pulse radiolysis (ca. 10 Gy in 200 ns) of solutions saturated with O_2/N_2 mixtures, containing tarloxotinib (1 mM) in methanol (●).
